# OnabotulinumtoxinA for chronic migraine treatment: 75% responder analysis from double-blind, randomized, placebo-controlled phase of PREEMPT

**DOI:** 10.1186/1129-2377-14-S1-P197

**Published:** 2013-02-21

**Authors:** D Dodick, HC Diener, C Turkel, R DeGryse, M Brin

**Affiliations:** 1 Mayo Clinic Arizona, USA; 2 University of Essen, UK; 3 Allergan, USA

## Introduction

Chronic migraine (CM) is a prevalent and disabling neurological disorder. OnabotulinumtoxinA is the only approved therapy specifically for CM. The results from randomized controlled trials often reflect, but rarely define, the spectrum of patient outcomes. The proportion of patients highly responsive to a therapy is an important endpoint and guide for clinicians and patients.

## Objective

To determine the proportion of patients who are highly responsive (75% responder rate) to therapy in 2 double-blind, placebo-controlled, parallel studies (PREEMPT 1 & 2).

## Methods

PREEMPT (two phase 3 studies: 24-week, double-blind, placebo-controlled, parallel-group phase, followed by 32-week, open-label phase) evaluated onabotulinumtoxinA for prophylaxis of headaches in CM (15 days/month with headache lasting 4 hours/day or longer). Patients were randomized (1:1) to onabotulinumtoxinA (155-195U) or placebo every 12 weeks. The proportions of patients with 75% decrease from baseline in frequency of headache days, headache episodes, migraine days, migraine episodes, moderate/severe headache days, and total cumulative hours of headache on headache days were analyzed.

## Results

Pooled analyses (onabotulinumtoxinA n=688, placebo n=696) demonstrated a statistically significant between-group difference favoring onabotulinumtoxinA in the proportion of patients who had a 75% reduction from baseline in headache days at Week 24 (22.8% onabotulinumtoxinA, 15.5% placebo; p=0.002). For all above headache symptom measures, a significantly greater proportion of onabotulinumtoxinA-treated than placebo-treated patients had 75% decreases from baseline.

## Conclusions

PREEMPT supports the efficacy and tolerability of onabotulinumtoxinA for the prophylaxis of headache in adults with CM.[1] These data demonstrate that onabotulinumtoxinA treatment results in a significant 75% reduction in multiple headache symptom measures and highly substantial efficacy for a subpopulation of patients studied.

## Support

Allergan, Inc.

## References

[B1] Dodick DW Headache 2010 14 6 921 63 10.1111/j.1526-4610.2010.01678.x 20487038

